# Bm86 midgut protein sequence variation in South Texas cattle fever ticks

**DOI:** 10.1186/1756-3305-3-101

**Published:** 2010-11-03

**Authors:** Jeanne M Freeman, Ronald B Davey, Lowell S Kappmeyer, Diane M Kammlah, Pia U Olafson

**Affiliations:** 1 USDA/ARS Knipling-Bushland United States Insects Research Laboratory, Kerrville, TX, USA; 2 USDA/ARS Cattle Fever Tick Research Laboratory, Edinburg, TX, USA; 3 USDA/ARS Animal Disease Research Unit, Pullman, WA, USA

## Abstract

**Background:**

Cattle fever ticks, *Rhipicephalus (Boophilus) microplus *and *R. (B.) annulatus*, vector bovine and equine babesiosis, and have significantly expanded beyond the permanent quarantine zone established in South Texas. Currently, there are no vaccines approved for use within the United States for controlling these vectors. Vaccines developed in Australia and Cuba based on the midgut antigen Bm86 have variable efficacy against cattle fever ticks. A possible explanation for this variation in vaccine efficacy is amino acid sequence divergence between the recombinant Bm86 vaccine component and native Bm86 expressed in ticks from different geographical regions of the world.

**Results:**

There was 91.8% amino acid sequence identity in Bm86 among *R. microplus *and *R. annulatus *sequenced from South Texas infestations. When South Texas isolates were compared to the Australian Yeerongpilly and Cuban Camcord vaccine strains, there was 89.8% and 90.0% identity, respectively. Most of the sequence divergence was focused in one region of the protein, amino acids 206-298. Hydrophilicity profiles revealed that two short regions of Bm86 (amino acids 206-210 and 560-570) appear to be more hydrophilic in South Texas isolates compared to vaccine strains. Only one amino acid difference was found between South Texas and vaccine strains within two previously described B-cell epitopes. A total of 4 amino acid differences were observed within three peptides previously shown to induce protective immune responses in cattle.

**Conclusions:**

Sequence differences between South Texas isolates and Yeerongpilly and Camcord strains are spread throughout the entire Bm86 sequence, suggesting that geographic variation does exist. Differences within previously described B-cell epitopes between South Texas isolates and vaccine strains are minimal; however, short regions of hydrophilic amino acids found unique to South Texas isolates suggest that additional unique surface exposed peptides could be targeted.

## Background

Vaccines that inhibit tick survival and impede transmission of the blood borne pathogens they vector are integral for improving both human and animal health. Vaccines against cattle fever ticks, the one host ticks that transmit bovine and equine babesiosis, are utilized in endemic countries such as Australia and Cuba [1]. These vaccines are not approved for use in the United States, and current eradication methods established by the United States Cattle Fever Tick Eradication Program rely on acaricide treatment of infested cattle and equids [2]. Recently, temporary quarantine blanket areas have been implemented in areas previously known to be free of cattle fever ticks [2]. Obstacles to existing strategies designed to control cattle fever ticks in the U.S include the development of acaricide resistance, transport of ticks from infested to uninfested pastures by wildlife hosts, and maintenance of ticks on currently infested pastures by wildlife hosts [2,3]. Each of these factors has contributed to the expansion of temporary quarantine blanket areas outside of the permanent quarantine zone in South Texas, resulting in the urgent need to implement new effective strategies against cattle fever ticks on both cattle and white-tailed deer. One such method involves the development and testing of vaccine candidate antigens. Vaccination of susceptible mammalian hosts is one tool with the potential to substantially reduce pasture tick prevalence and decrease host exposure to ticks and the pathogens they transmit.

Commercially available vaccines against cattle fever ticks that are approved for use outside of the United States, including Gavac^® ^(Heber Biotec; Havana, Cuba), TickGARD (Hoechst Animal Health; Australia), and TickGARD^PLUS ^(Intervet Australia; Australia), are based on the recombinant form of the concealed midgut antigen, Bm86. The efficacy of Bm86 based vaccines against *R. microplus *and *R. annulatus *infestations is highly variable given the wide variety of experimental conditions and efficacy parameters under which they have been tested. Outside the U.S, vaccine efficacy following immunization with Bm86 and challenge with *R. microplus *has ranged from 0 to 91%, while efficacy against *R. annulatus *is reported to be above 99% [1,4-8]. A possible explanation for this variation in vaccine efficacy is divergence in amino acid sequence between the recombinant Bm86 in vaccines and Bm86 in ticks found in various geographical regions. Based on a calculation of mutation fixation index applied to a sequence fragment containing amino acids 539-573 of Bm86 from Australian, Mexican, Cuban, Venezuelan, and Argentine strains, Garcia-Garcia *et al *[9] suggested there was an inverse correlation between the vaccine efficacy and sequence variation within the Bm86 locus. Specifically, it was concluded that an amino acid sequence divergence of greater than 2.8% would result in a decrease in vaccine efficiency [9]. However, despite a high degree of sequence conservation in the Bm86 gene between sequenced *R. microplus *strains, vaccination with Gavac is not as effective against several Argentine and Mexican strains [6]. Other possible explanations for variation in vaccine efficacy related to the vector itself include differences in expression levels during targeted tick stages, existence of conformational epitopes not covered by antigen presentation in vaccine preparations, or differences in the quantity of blood and therefore anti-Bm86 antibody imbibed by various tick species [10].

Although antibody titer has been correlated with protection following vaccination with Bm86, little is known about specific protective epitopes, and the presence of sequence variation in these epitopes within a South Texas tick population. The objective of this research was to identify the presence or absence of Bm86 sequence variation found in larval progeny of *R. microplus *and *R. annulatus *obtained from cattle and white-tailed deer hosts within the permanent and blanket quarantine zones, as well as outbreak strains established in colony over several generations. Predicted B-cell epitopes and surface-exposed regions found within Bm86 sequences of South Texas isolates were then compared to those in isolates used to develop commercially available vaccines.

## Results and Discussion

**Figure 1:**
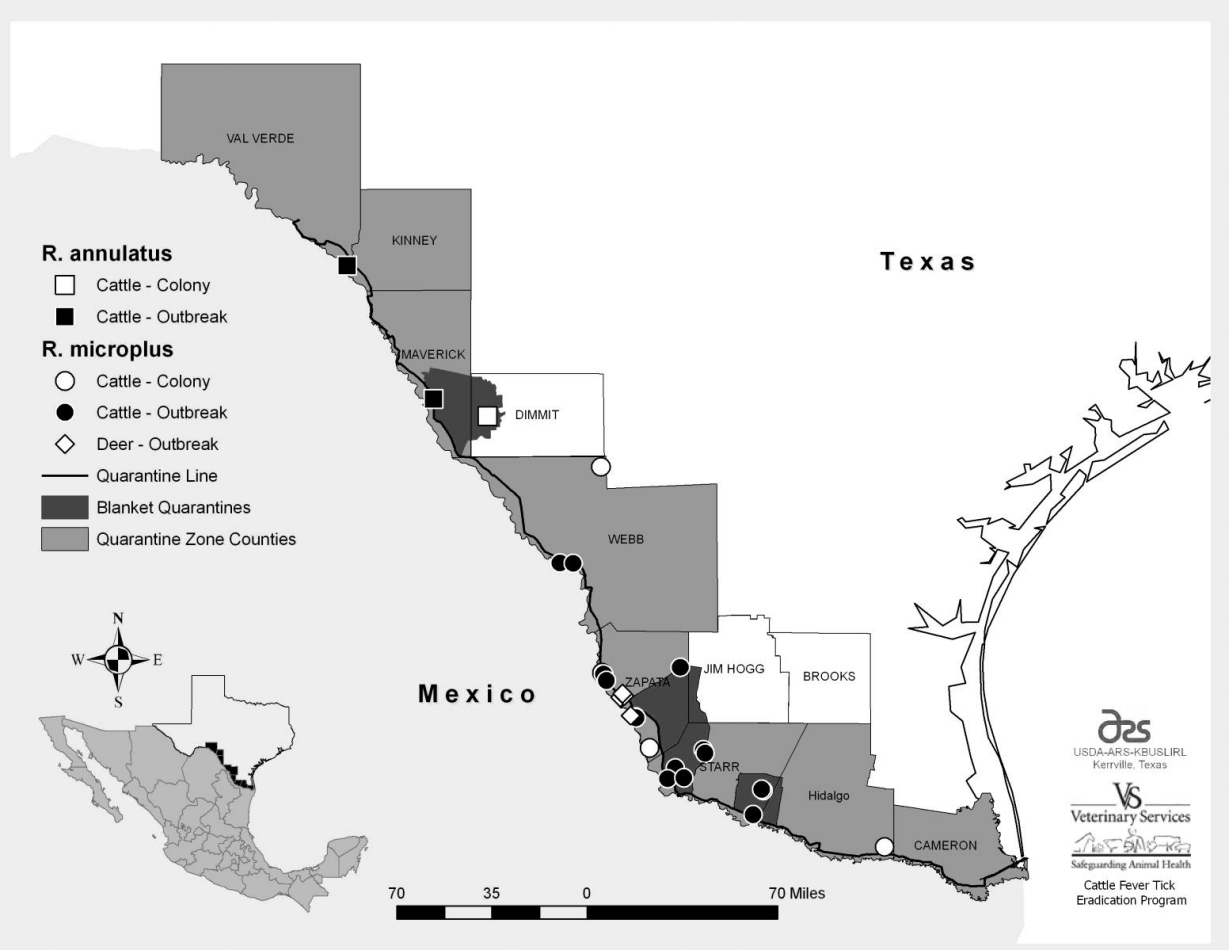
**Map of temporary blanket and permanent quarantine zones**. Bm86 sequences obtained from larval colony or outbreak samples are represented by squares (*R. annulatus*, cattle host), circles (*R. microplus*, cattle host), or diamonds (deer hosts). The blanket quarantine zone is shaded in dark grey and the permanent quarantine zone is shaded in light grey.

Sequence variation in the tick midgut surface protein Bm86 is one hypothesis for the variability in efficacy of Bm86 based vaccines against cattle fever ticks. In order to evaluate the potential use of a Bm86 based immunogen against cattle fever ticks in South Texas, the full length *Bm86 *coding region was sequenced from the larval stage of isolates recently obtained from within the both the permanent and temporary quarantine zones, so as to represent the current repertoire of *Bm86 *sequences existing in the field. Larval isolates from 27 different collections were selected for sequencing of the *Bm86 *coding sequence (Figure 1; Table 1). The number and location of isolates selected reflects the proportion of current outbreaks in their respective counties, as well as the number of female ticks submitted through the CFTEP that were available for oviposition. This number of isolates is also comparable to that used in previous descriptions of Bm86 geographic sequence variability [11]. The majority of documented infestations in South Texas are *R. microplus*, and this is reflected in the number of isolates sequenced: 24 *R. microplus *and 3 *R. annulatus *(Table 1). Twenty-two of 27 isolates sequenced were obtained from cattle hosts, reflecting the smaller number of opportunities to collect and evaluate ticks from white-tailed deer hosts.

**Table 1:** Larval isolates utilized for Bm86 sequencing and resulting sequence groups.

County and isolate number	**Date of submission**^**a**^	Tick species	**Tick host**^**b**^	Source of larvae	**Number of females used in oviposition**^**c**^	**Larval generation**^**d**^
Dimmit 1	8/10/07	*R. annulatus*	bovine	colony	160	F6

Hidalgo 1	8/3/04	*R. microplus*	bovine	colony	17	F17

Kinney 1	2/10/09	*R. annulatus*	bovine	outbreak	33	F1

Maverick 1	10/22/09	*R. annulatus*	bovine	outbreak	33	F1

Starr 1	2/17/09	*R. microplus*	bovine	outbreak	115	F1

Starr 2	3/24/09	*R. microplus*	bovine	outbreak	19	F1

Starr 3	1/16/09	*R. microplus*	bovine	outbreak	30	F1

Starr 4	3/24/09	*R. microplus*	bovine	outbreak	63	F1

Starr 5	5/20/09	*R. microplus*	bovine	outbreak	80	F1

Starr 6	4/20/09	*R. microplus*	bovine	outbreak	3	F1

Starr 7	4/3/09	*R. microplus*	bovine	outbreak	3	F1

Starr 8	4/15/09	*R. microplus*	bovine	outbreak	50	F1

Webb 1	10/16/01	*R. microplus*	bovine	colony	55	F29

Webb 2	1/26/09	*R. microplus*	bovine	outbreak	2	F1

Webb 3	12/2/09	*R. microplus*	bovine	outbreak	38	F1

Zapata 1	8/20/99	*R. microplus*	bovine	colony	130	F45

Zapata 2	1/7/05	*R. microplus*	bovine	colony	300	F15

Zapata 3	2/5/09	*R. microplus*	bovine	outbreak	29	F1

Zapata 4	4/20/09	*R. microplus*	bovine	outbreak	30	F1

Zapata 5	10/22/09	*R. microplus*	bovine	outbreak	195	F1

Zapata 6	7/15/09	*R. microplus*	bovine	outbreak	34	F1

Zapata 7	12/9/09	*R. microplus*	deer	outbreak	17	F1

Zapata 8	12/2/09	*R. microplus*	bovine	outbreak	8	F1

Zapata 9	1/21/10	*R. microplus*	deer	outbreak	3	F1

Zapata 10	1/13/10	*R. microplus*	deer	outbreak	12	F1

Zapata 11	12/17/09	*R. microplus*	deer	outbreak	2	F1

Zapata 12	1/21/10	*R. microplus*	deer	outbreak	30	F1

^a^Date that adult female was submitted to CFTRL in Mission, TX.

^b^Deer = white-tailed deer.

^c^The number of females from which eggs were pooled to produce the larval progeny used in Bm86 sequencing.

^d^*Rhipicephalus *maintained in colony at CFTRL after the initial outbreak are designated "colony" along with the generation of larvae from which Bm86 was sequenced. Larvae obtained from field collected adult female *Rhipicephalus *are listed as F1 "outbreaks".

**Figure 2:**
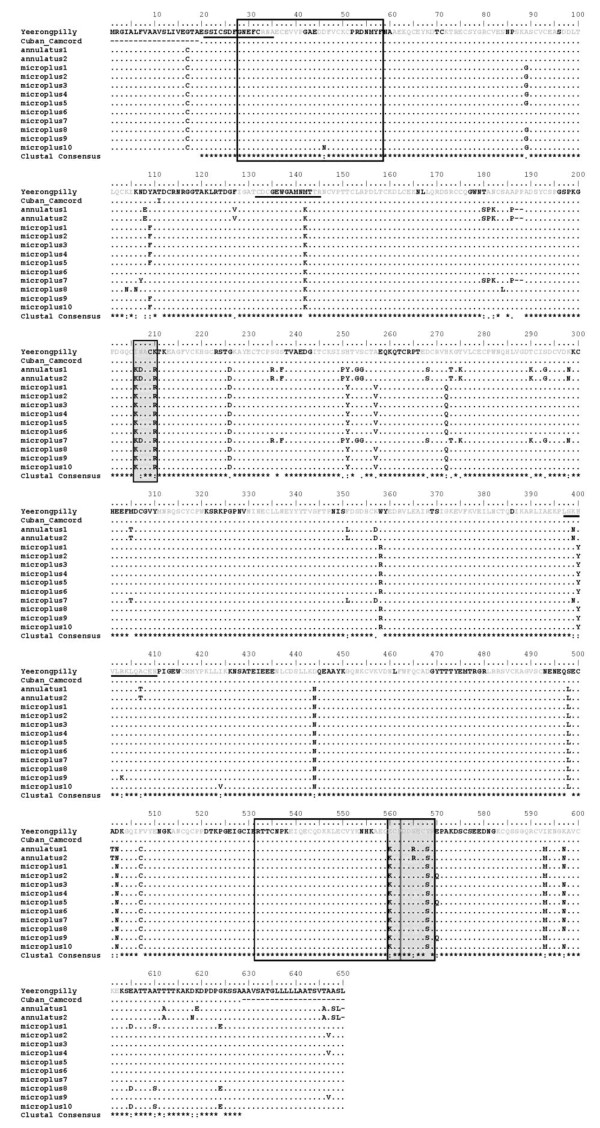
**Amino acid alignment of Yeerongpilly (Australian strain), Cuban Camcord (Cuban strain used in Gavac vaccine)**. Linear B-cell epitopes previously identified by Odongo *et al. *are boxed; synthetic immunogenic peptides previously identified by Patarroyo *et al. *are underlined; antigenic peptides predicted using the method of Kolaskar and Tongaonkar (Canales 2009) are in grey font; hydrophilic regions (identified using Hopp & Woods algorithm in BioEdit sequence alignment editor; window size of 5) of south Texas isolates not found in Yeerongpilly or Camcord isolates are shaded.

Bm86 from 27 South Texas isolates separated into 12 distinct groups based on amino acid sequence identity (Figure 2). The number of isolates per group varied between 1 and 9 (Table 2). Overall, there was 91.8% amino acid sequence identity in *Bm86 *among all of the isolates sequenced. Stronger similarities existed when comparing amino acid sequences within species, with 93.7% identity within all *R. microplus *isolates sequenced and 99.7% identity between the 3 *R. annulatus *isolates sequenced. Interestingly, 3 of the 5 Bm86 sequences obtained from larval strains that were maintained in colony over several generations, (Tables 1, 2) had 100% sequence identity to field collected isolates. This is consistent with the findings of Garcia-Garcia *et al. *(1999) where field derived strains from Argentina were identical to the Argentine laboratory reared strain A.

**Table 2:** Identical Bm86 sequences grouped by county and isolate number.

	**Isolate county**^**2**^** and number^3^**						
**Designated Bm86 sequence group**^**1**^	Starr	Zapata	Hidalgo	Webb	Dimmit	Maverick	Kinney
*R. microplus *1	3, 4, 6, 7, 8	2, 6, 7		1			

*R. microplus *2	5	3, 4, 8, 9		2, 3			

*R. microplus *3		5					

*R. microplus *4		10					

*R. microplus *5		1					

*R. microplus *6		12					

*R. microplus *7			1				

*R. microplus *8	1						

*R. microplus *9	2						

*R. microplus *10		11					

*R. annulatus *1					1	1	

*R. annulatus *2							1

^1^groups consist of larval isolates having 100% identical nucleotide sequences.

^2^county where adult female cattle fever ticks were obtained

^3^isolate number used in Table 1.

Consistent with the Yeerongpilly Bm86 sequence [4], all South Texas Bm86 sequences were predicted to contain a glycosyl-phosphatidylinositol anchoring signal sequence. In addition, epidermal growth factor repeats consisting of a 6 cysteine residue pattern have been previously described in the Yeerongpilly strain [4] and in various homologs of Bm86 [12]. There is one additional cysteine residue present in the signal peptide of South Texas isolates (residues 17) that is not present in the Yeerongpilly strain (the Camcord sequence in this region is not available in GenBank).

According to Garcia-Garcia *et al. *(1999), there is an inverse correlation between vaccine efficacy and sequence variation in Bm86, with variations of greater than 2.8% being the most likely to produce lower efficacy. Therefore, a comparison between Bm86 from South Texas isolates and Bm86 from vaccine strains was evaluated. In our comparisons, we utilized Bm86 from the Cuban Camcord strain of *R. microplus*, which is the component of commercially available Gavac [13], and the Australian Yeerongpilly strain of *R. microplus *[4]; GenBank Accession M29321.1), which is the component of commercially available TickGard and TickGARD^PLUS ^. The level of sequence identity among all aligned South Texas *R. microplus *isolates (93.7%) is lower when compared to the identity between the Cuban Camcord and Australian Yeerongpilly strains (98.4%), suggesting that there is significantly greater diversity within the Bm86 gene in this South Texas population. An alignment of all full-length South Texas isolates was then compared to vaccine strains, revealing 89.8% and 90.0% amino acid sequence identity between Bm86 from South Texas isolates and the full-length Yeerongpilly and partial length Camcord strains, respectively. When *R. annulatus *isolates are removed from the comparison, there is 8.3% sequence variation between *R. microplus *isolates from South Texas and the Yeerongpilly strain. These percent differences are consistent with previously published data by Garcia-Garcia *et al. *(1999), where Yeerongpilly differed from Mexican and Argentine *R. microplus *field isolates by 5.7 - 8.6%.

Amino acid sequence variation from South Texas isolates was found throughout the entire length of Bm86, with a region of concentrated sequence variation spanning amino acids 206-298 (Figure 2). Four peptides predicted to be antigenic in the Cuban Camcord strain by Canales *et al *[13] fall into this region. Three of these peptides had amino acid differences in South Texas isolates (between 2 and 6 differences are observed within a peptide) which in general contributed to a more hydrophobic nature in these specific regions (Figure 2.) However, the region of amino acids 206-210 of South Texas isolates was hydrophilic compared to the same region in Cuban Camcord and Yeerongpilly isolates (shaded in Figure 2). All *R. annulatus *and *R. microplus *isolates sequenced from South Texas have a lysine (K) in place of an isoleucine (I) at position 206) followed by an aspartic acid (D) (in both *R. annulatus *isolates and the *R. microplus *group 7 sequence) in place of an asparagine (N). Another region spanning amino acids 560-570 is also more hydrophilic in South Texas isolates when compared to the vaccine strains analyzed (Figure 2). It is not known if these differences will affect the presentation of these epitopes to the immune system following vaccination with recombinant protein. It can be hypothesized, however, that a recombinant form of Bm86 reflecting amino acid differences apparent in South Texas isolates could elicit an immune response to a different repertoire of epitopes versus that elicited by those within vaccine strains.

Quantification of antibody titers against Bm86 has been the main method of measuring the bovine immune response following vaccination [8,14]. Antibody titers have been shown to correlate with protection, however, the epitopes found in Bm86 that induce protective immune responses in cattle are not extensively characterized. Linear B-cell epitopes previously identified by Odongo *et al *[15] and three synthetic peptides shown to elicit protective antibody responses characterized by Patarroyo *et al *[16] and Peconick *et al. *[17] are indicated in Figure 2. When compared to Yeerongpilly and Camcord, there was only one amino acid difference within the linear B-cell epitopes present in South Texas isolates, a lysine (L) in place of glutamic acid (E) at position 560. Peconick et al. [17] evaluated three immunogenic epitopes in the synthetic vaccine SBm7462 based on the Australian Yeerongpilly strain. There were 4 total amino acid differences observed within these three previously described peptides shown to induce protective immune responses in cattle. There are no differences within the first peptide. The second peptide, located at amino acids 132-145, contains a lysine (L) in all Texas isolates in place of a methionine (M) in vaccine strains, while there is a substitution of a tyrosine (T) in place of a histidine (H) in the *R. microplus *strains within the third peptide located at amino acids 398-411. *R. annulatus *sequences have two amino acid differences within this peptide, an asparagine (N) in place of a lysine (L) and threonine (T) in place of an alanine (A).

In the Cuban Camcord strain, Canales *et al *(2009) identified B-cell epitopes predicted based on physiochemical properties of amino acids such as hydrophilicity and presence in epitopes of other species, based on the method of Kolaskar and Tongaonkar [13,18]. These peptides are highlighted in grey in Figure 2. Several differences occur within these peptide sequences when comparing the Cuban Camcord strain with South Texas isolates, the majority of which are localized the region of amino acids 206-298. Differences in these predicted B-cell epitopes occur in both *R. microplus *and *R. annulatus *isolates. It is not known if these amino acid differences have any effect on antibody response following vaccination. However, the selection of epitopes that are specific to South Texas field collected ticks will be potentially useful in vaccine design. Because of the sequence diversity found within Bm86 from South Texas isolates, it is conceivable that a vaccine based on this antigen should contain several of the predicted variant epitopes in order to be effective.

## Conclusions

Amino acid sequence variation among South Texas cattle fever tick isolates was apparent throughout the full length Bm86 protein but was concentrated in amino acids 206 - 298. Several variations within previously described predicted epitopes of vaccine strains have been identified. *In vivo *immunogenicity trials are necessary to further determine which epitopes relevant to South Texas isolates will be the most effective immunogens.

## Materials and methods

### Tick isolates

Adult female cattle fever ticks were obtained through the Cattle Fever Tick Eradication Program established and maintained by USDA-APHIS-VS. Larval isolates were selected from outbreak submissions based on availability and preference was given to isolates obtained from counties with the highest proportion of current outbreaks (Starr and Zapata counties). Oviposition by female ticks occurred at the USDA-ARS Cattle Fever Tick Research Laboratory (CFTRL) in Mission, TX. The total number of females from which eggs were collected and pooled to produce larval progeny was recorded. Resulting larval progeny were either maintained in colony at the CFTRL (5 colony strains) or aliquots were frozen for RNA isolation (22 isolates). The outbreak isolates were collected between January 2009 and January 2010 from infested cattle or white-tailed deer.

### Total RNA isolation and cDNA synthesis

Approximately 100 larvae from each isolate were placed in RNA-later-ICE solution (Ambion, Austin, TX, USA) and stored at -20C. Total RNA was isolated from larval samples following the protocols described in the ToTally RNA Kit™(Ambion). Following treatment with Turbo DNA-*free*™(Ambion), first-strand cDNA was synthesized using SuperScript II™First-Strand Synthesis System for RT-PCR (Invitrogen, Carlsbad, CA, USA). The cDNA (1 μl) was subsequently used as template to amplify full length Bm86 (2023 bp) using primers designed to anneal to conserved regions: forward primer 5'ATGCGTGGCATCGCTTTGTT 3' and reverse primer 5'-GGTGTTCGATGTAAGCGTGATG-3'. PCR was performed using Platinum Taq (Invitrogen) under the following conditions: 94°C for 1:30, 35 cycles of 94°C for 30 seconds, 55°C for 30 seconds, and 72°C for 2:00, followed by a final extension at 72°C for 4:30. Amplified products were gel-purified using QIAquick^® ^Gel Extraction Kit and cloned using pCR^®^4-TOPO TA Cloning^® ^Kit for Sequencing (Invitrogen). Clones were PCR screened using 5 Prime Hot Master Mix along with M13F and M13R primers and plasmid DNA's isolated using FastPlasmid^® ^Mini Kit (5 Prime, Gaithersburg, MD, USA).

### Bm86 sequencing and analysis

A minimum of five clones from each strain were sequenced using BigDye^® ^Terminator version 3.1 chemistry (Applied Biosystems, Carlsbad, CA, USA). Forward and reverse M13 sequencing primers were used in addition to the following five sequencing primers designed within the Bm86 gene to obtain double strand coverage of the full-length gene: Bm86for685, 5'- GACGAAAGAAGCTGGGTT-3';

Bm86rev605, 5'-CCAGGAGAGCAATAGGAGTC-3'; Bm86for1650, 5'- GTACCACATGCAACCCTAAA-3'; Bm86 internal reverse, 5'-TTTCTCTGCTATGAGTCTTGCC-3'

Bm86 internal forward, 5-'ATCGACAAAGCTGCTATTGTCC-3'. A nucleotide consensus sequences was obtained from each isolate and then translated using the translate tool on the ExPASy Proteomics server http://www.expasy.org/tools/dna.html. Amino acid sequences from each isolate were then aligned using ClustalW2 and grouped according to identity using Vector NTI Advance 11 (Invitrogen) and BioEdit Sequence Alignment Editor version 7.0.5.3 [19].

GPI anchor prediction was performed using the online GPI-anchor prediction program PredGPI http://gpcr2.biocomp.unibo.it/gpipe/index.htm. The method of Hopp & Woods (using a window size of 5) was used to predict hydrophilic regions in BioEdit sequence alignment editor.

Nucleotide sequence data reported in this paper are available in the GenBank™ database under accession numbers HQ014385, HQ014386, HQ014387, HQ014388, HQ014389, HQ014390, HQ014391, HQ014392, HQ014393, HQ014394, HQ014395, HQ014396, HQ014397, HQ014398, HQ014399, HQ014400, and HQ014401.

## Competing interests

The authors declare that they have no competing interests.

## Authors' contributions

JMF designed and performed molecular biology experiments, analyzed the data, and wrote the manuscript. RBD provided data on the isolates selected for sequencing. LSK advised on molecular biology and bioinformatics data analysis and interpretation. DK obtained coordinates for and generated the GIS map. PUO performed molecular biology experiments and advised on data interpretation. All authors reviewed the manuscript before submission.
